# Aflatoxin, microbial contamination, sensory attributes, and morphological analysis of pistachio nut coated with methylcellulose

**DOI:** 10.1002/fsn3.2212

**Published:** 2021-03-06

**Authors:** Zeinab Moslehi, Abdorreza Mohammadi Nafchi, Marzie Moslehi, Shima Jafarzadeh

**Affiliations:** ^1^ Food Science and Technology Department Damghan Branch Islamic Azad University Damghan Iran; ^2^ Food Biopolymer Research Group Food Technology Division School of Industrial Technology Universiti Sains Malaysia Penang Malaysia; ^3^ Engineering Department Ayatollah Amoli Branch Islamic Azad University Amol Iran

**Keywords:** aflatoxin contamination, methylcellulose coating, pistachio nuts, sensory attributes

## Abstract

Pistachio is a nut with high consumption that could be affected by aflatoxin contamination, which affects the consumption market; therefore, broad studies seem to be necessary for this area. In the current study, pistachio nuts (Abbasali variety) were coated with different concentrations (0.1%, 0.5%, 1%, and 2%) of methylcellulose (MC) by immersion method and then stored in the incubator (25°C) for four months. The inhibitory effect of hydrocolloid coating on microbial (mold, yeast, and total count) and aflatoxin (B1, B2, G1, G2, and total aflatoxin) contamination, as well as sensory attributes (flavor, color, crispiness, aroma, and total acceptability), was investigated during storage periods. Results showed that the storage period had a significant effect on yeast, mold, and total count. HPLC analysis results showed that coating with MC had a significant inhibitory effect on aflatoxin contamination, and the highest amount of aflatoxin contamination was related to the control sample (3.5%). All samples except sample coated with MC 0.5% had appropriate total acceptability. Regarding the inhibitory effect of MC edible coating on aflatoxin contamination, its application on pistachio nut could be a promising approach to control the fungus infection and reduce aflatoxin production in coated pistachio.

## Graphical abstract

The appropriate coating of pistachio nut is an effective step for reducing the aflatoxin amount. The storage of the pistachio nut period had a significant effect on yeast, mold, and total count.

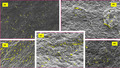

## 1 INTRODUCTION

Pistachio is one of the most popular nuts globally (Wu & Xu, 2020) grown in different countries such as Iran, the USA, Turkey, Syria, Italy, and Greece (Catalán et al., 2017). It contains high amounts of phenolic compounds with unique efficiency and high antioxidant potential (Wu et al., 2018). However, this valuable product is very susceptible to rancidity and molds' contamination (Holakouie Naieni et al., 2020). Contamination by storage pests is a significant problem faced with this product. Mold contamination in crops leads to the formation of critical secondary metabolites named mycotoxins, which are secondary metabolites of different fungus including *Aspergillus flavus, Aspergillus parasiticus, Aspergillus tamarri, Aspergillus bombycitts,* and *Aspergillus nomius*. These funguses produce different aflatoxins including B1, B2, G1, and G2. The metabolites mentioned above increase the risks of cancer and mutation in humans and animals (Ortega‐Beltran et al., 2019). Harvest period, transportation, storage condition, and preservation method are the most important factors that affect contamination of pistachio nuts by mold spores (Hadavi et al., 2017).

Today demand for using natural polymers to produce edible films for food packaging increased due to their environmental‐friendly properties and biodegradability (Chong et al., 2019; Esfahani et al., 2020; Jafarzadeh & Jafari, 2020; Mei et al., 2020; Mohammadi Nafchi et al., 2017). Edible coatings are a thin layer of the natural substances surrounding the food surfaces of products (Ekramian et al., 2021; Kazemi et al., 2020; Mousavian et al., 2021). They act as a barrier agent and consequently protect the food product from undesirable changes in flavor, texture, and apparent properties (Ahmad et al., 2016; Garavand et al., ,^2017^, ^2020^). These coatings can be prepared by the immersion method as a continuous layer that covers the product surface (Hermawan et al., 2019; Jafarzadeh et al., 2017, 2019). Polymeric compounds such as proteins, polysaccharides, lipids, and their derivatives have been used for preparing edible films. Methylcellulose (MC) is one of the most common polysaccharides used as an edible coating (Moosavian et al., 2017). Cellulose is a plant constituent and a source of complicated carbohydrates in the world. These substances act as an appropriate moisture and oxygen barrier due to their flexibility, transparency, and high‐fat resistance. Several studies have been done on the edible coating on aflatoxin contamination in different nuts (Kazemian‐Bazkiaee et al., 2020; Shirdeli et al., 2020; Tavakolipour et al., 2020).

To the best of our knowledge, there have been limited studies on the effect of MC coating on pistachio nut contamination by aflatoxin. Therefore, this study aimed to develop MC coating, and the impact of prepared coating on morphological, microbial, and sensory properties of pistachio nuts during four months of storage was investigated.

## 2 MATERIALS AND METHODS

### 2.1 Materials

Abbasali pistachio nut samples were obtained from the local market (Damghan, Iran). All other materials were of analytical grade.

### 2.2 Preparation of methylcellulose solution

The coating was performed by the immersion method. For this purpose, pistachio kernels were randomly divided into 1 kg packages. In a tube containing distilled water, MC at different concentrations (0.1%, 0.5%, 1%, and 2%) was gradually added. The MC was solved entirely in water after 30–45 min. After exiting air bubbles, the MC solution was ready for coating.

### 2.3 Coating procedure

For the coating process at each concentration, 1 kg pistachio kernels was immersed in the MC solution and stirred for 5 min. The coated samples were stored in an oven (25°C) for 3 days. Dried coated samples were packed in polyethylene bags and kept at an incubator (25°C) for 4 months (Tesfay et al., 2017). Pistachios without MC edible coating were used as control sample (uncoated).

### 2.4 Aflatoxin assay

**FIGURE 1:**
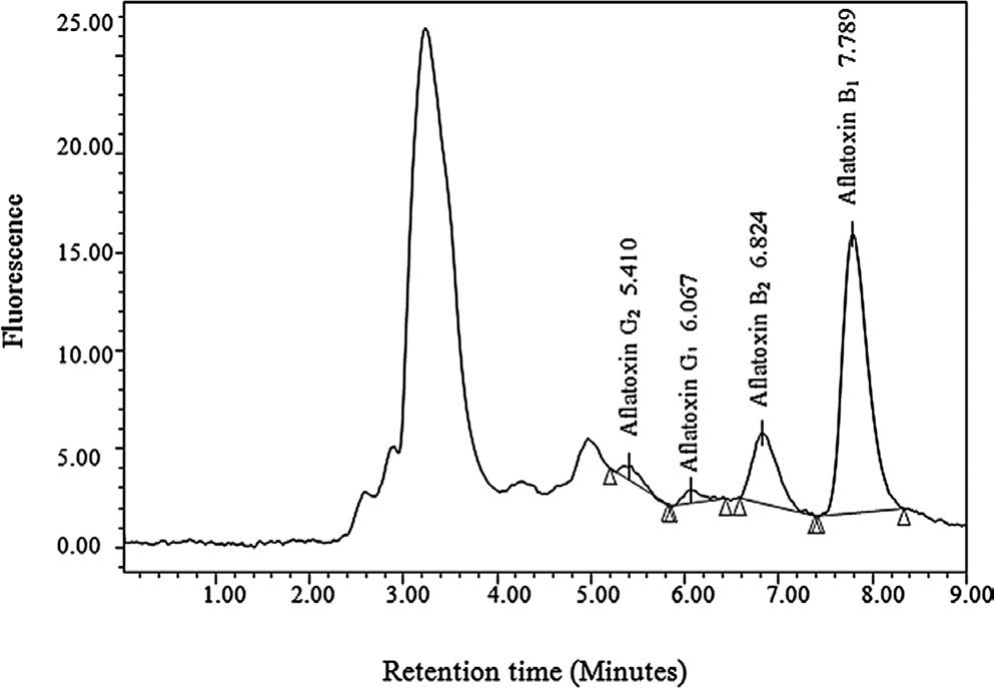
Obtained pick for aflatoxin B1, aflatoxin B2, aflatoxin G1, and aflatoxin G2 by immunoaffinity column high‐performance liquid chromatography

A capillary waters 2,475 chromatography, column c18 (Novapack) equipped with multi‐ and fluorescence detector was used in aflatoxin measurement. First, 50 g pistachio sample was milled with 5 g salt, 100 ml hexane, and 200 ml methanol and then centrifuged. The obtained supernatant (20 ml) was then filtered, mixed with 130 ml water, and homogenized for 5 min. The homogenate slurry was filtered by filter paper using glass fiber, and 70 ml of the filtrate was fed to the Aflatest column. The column was eluted with 15 ml of phosphate buffer and 15 ml of distilled water. Finally, aflatoxin was removed by passing methanol through the column, and 1.5 ml of distilled water was added (Karami‐Osboo & Mirabolfathi, 2017). Figure 1 shows the obtained pick for aflatoxin B1, aflatoxin B2, aflatoxin G1, and aflatoxin G2 by high‐performance liquid chromatography.

### 2.5 Microbial assay

Total count, mold, and yeast assays were performed according to the national standard, ISO 8442 and ISO 21527‐2 respectively (ISO 21527–2. 2008, ISO 8443:2003).

### 2.6 Sensory attributes

Sensory characteristics, including color intensity, aroma and flavor, crispiness, and total acceptability, were evaluated by 12 panelists at the end of the storage period. A 5‐point hedonic scale (1 = very bad, 2 = bad, 3 = mediate, 4 = good, and 5 = very good) was used. From each treatment, six pistachio nut samples were placed in special colorless plastic containers and offered to each assessor to evaluate four main sensory attributes: color, aroma, flavor, and overall acceptability (Delvarianzadeh et al., 2020).

### 2.7 Imaging by electron microscopy via SEM

**FIGURE 2:**
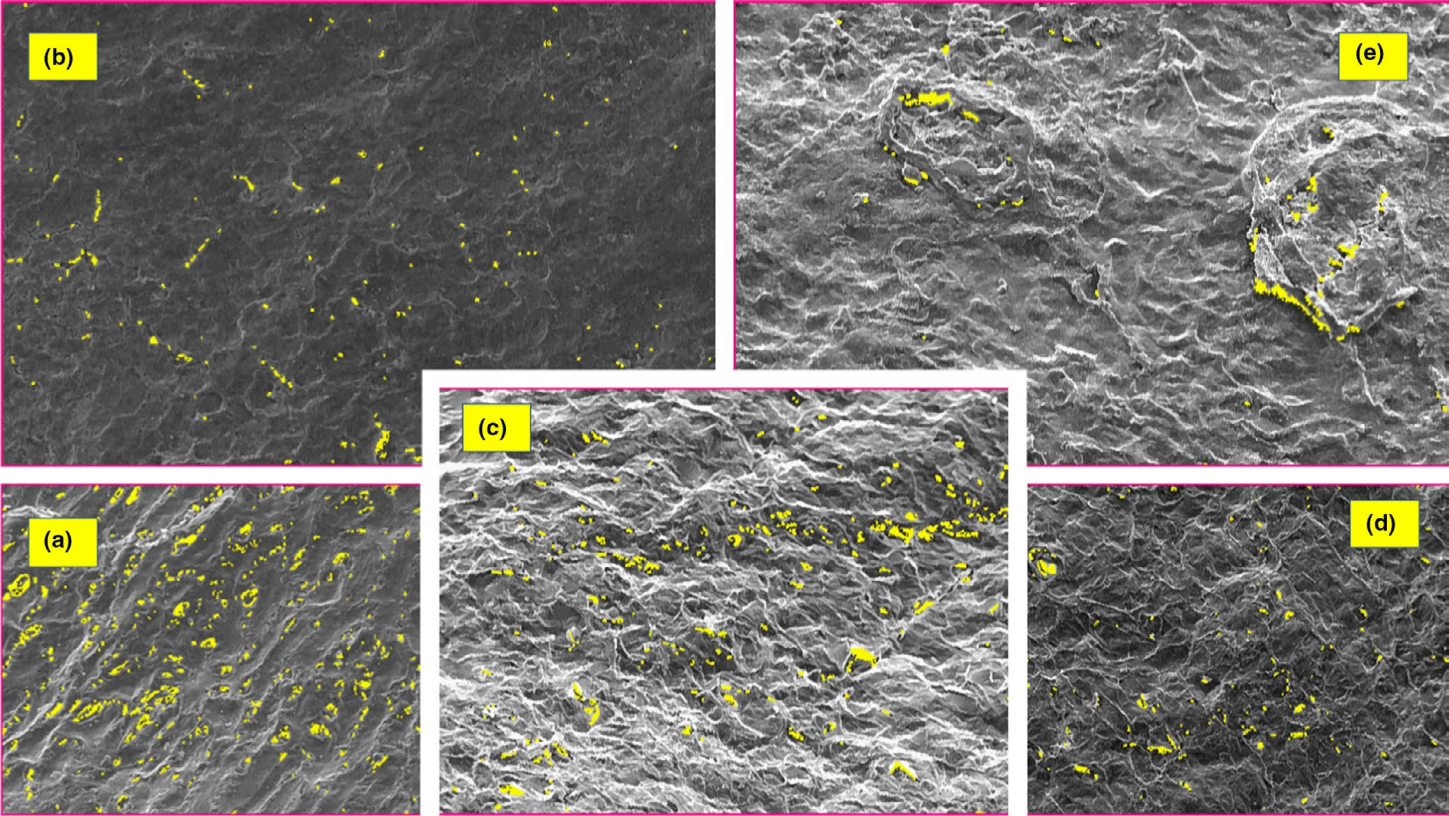
Percentage of porosity. (a) Control sample, (b) sample coated with 0.1% methylcellulose, (c) sample coated with 0.5% methylcellulose, (d) sample coated with 1% methylcellulose, and (e) sample coated with 2% methylcellulose

SEM (XL30, Philips Co.) was used to prepare images, and gold coating was done using sputter coater, BAL‐TEC SCD 500 model. To study coating characteristic of each sample, two sets of photographs were taken from the surface and a cross section of pistachio nuts. To prepare fixed samples, quick freezing–vacuum drying was used. For this purpose, samples were first immersed in liquid nitrogen, followed by breaking and vacuum drying. To obtain conductivity, samples were gold‐coated and underwent SEM. Taking a photograph from the surface does not need preparation, such as for cross section. Regarding that porosity determination in imaging software is based on resolution degree, images should be selected so that no other factors but porosity be measured. Therefore, inverted electron images (BSE) and secondary electron images should be taken from a certain point to be identified by comparing them with real porosities. On the other hand, color change in inverted electron images is due to phase change and the presence of porosity (Figure 2). Therefore, to obtain inverted electron images, the sample surface should not be uneven. Since the section surface of pistachio, the sample is not even (to obtain an even sample, its surface should have emerged polished). Since porosities are well detectable in secondary electron images, images were obtained by the secondary electron detector (Table 1).

**TABLE 1:** The size of coating porosity

Sample code	Porosity percent
Control sample	4.3%
Sample coated with 0.1% CMC	0.6%
Sample coated with 0.5% CMC	1.9%
Sample coated with 1% CMC	1%
Sample coated with 2% CMC	0.6%

### 2.8 Statistical analysis

To investigate the effect of different MC coating concentrations on the quality of stored pistachio nut samples, frequent measurements were done at different time intervals (0, 2, and 4 months) of the storage period. This design was used to investigate the significant difference in microbial and aflatoxin parameters (total count, yeast, and mold count). A completely randomized design (CRD) and the Duncan multiple‐range tests were used for comparing the quality attributes of coated and uncoated (control) samples during the storage period (0, 2, and 4 months of storage). All the experiments were performed at three replications, and SAS software (9.1) was used for statistical analysis.

## 3 RESULTS AND DISCUSSION

### 3.1 Aflatoxin

#### 3.1.1 Aflatoxin B1 and B2

As shown in Table 2, the results stated that the amount of aflatoxin B1 in the control sample was increased (from 0.47 to 1.85 μg/kg) after four months of storage. However, no significant difference was observed between uncoated (controls) and coated samples (except for coated samples with 2% MC) in terms of aflatoxin B1 content. After four months of storage, aflatoxin B1 content in the coated sample with 0.5% and 0.1% MC was decreased to an undetectable amount. Based on Table 2, the amount of aflatoxin B2 in the control sample was unchanged after four months of storage. In coated samples with 2% MC solution, the amount of aflatoxin B2 was increased during the storage period. There was no significant difference in the amount of aflatoxin B2 between control and coated samples (except for coated samples with 2% MC solution). During four months of storage, the amount of aflatoxin B2 in coated samples with 0.1%, 0.5%, and 1% MC was reduced to an undetectable amount. Similarly, Tavakolipour et al. (2011) investigated the inhibitory effect of coated pistachio based in WPC coating with thyme on aflatoxin production. The results showed that the concentration of WPC coating higher than 5,000 ppm could prevent aflatoxin production in pistachio. Moreover, Mohammadi et al. (2020) reported that the coated pistachio with CMC‐gelatin and *Dianthus barbatus essential oil* (DbE) showed no aflatoxin growth in three concentrations of 300, 450, and 600 ppm of DbE, while the uncoated pistachio showed a mild growth.

**TABLE 2:** Aflatoxin and microbial contamination of pistachio nuts as a function of coating

Treatment	B1	B2	G1	G2	Total	Mold and yeast (1000/g)	Total count (10^5^/g)
Control sample	1.85	0.09	1.72	0.08	3.74	466.70	3,000
Coated with 0.1% CMC	nd	nd	1.01	nd	1.01	166.70	1,000
Coated with 0.5% CMC	nd	nd	nd	nd	nd	0	0
Coated with 1% CMC	1.02	nd	1.74	nd	1.76	0	0
Coated with 2% CMC	0.91	0.16	1.51	0.22	2.80	0	0

Abbreviations: nd, not detected.

#### 3.1.2 Aflatoxin G1 and G2

According to Table 2, the amount of aflatoxin G1 in the control sample was increased from 0.4 to 1.72 μg/kg after 4 months of storage. The amount of aflatoxin G1 was increased in coated samples with 1% and 2% MC solution. There are no significant differences between coated samples with 0.1% and 0.5% MC solution in terms of aflatoxin G1. After 4 months of storage, the amount of aflatoxin G1 in the coated sample with a 0.5% and 0.1% MC solution was reduced to undetectable amounts.

The amount of aflatoxin G2 remained constant in the control sample after 4 months of storage (Table 2), whereas the amount of the aflatoxin G2 was increased in coated samples with 2% MC solution. There was no significant difference between control and coated samples in aflatoxin G2 (except for coated sample with 2% MC solution). During four months, the storage of the amount of aflatoxin G2 in coated samples with 0.1%, 0.5%, and 1% MC solution was reduced to an undetectable limit.

Tavakolipour et al. (2020) studied the impacts of coating pistachio kernels with WPC mixtures and selected plant extracts (sage, cumin, and Shirazi thyme) on the inhibition of aflatoxin during storage. The results exhibited that while the Shirazi thyme concentration increased in WPC coating, the aflatoxin production reduced. Besides, no G1 aflatoxin and G2 aflatoxin were found in pistachio‐coated samples when the amount of Shirazi thyme, sage, and cumin reached 5,000, 4,500, and 6,500 ppm, respectively. The sage extract had the highest preventing potential for aflatoxin generation compared with Shirazi thyme and cumin extracts.

#### 3.1.3 Total aflatoxin

As can be seen from Table 2, there is a significant difference between the coated sample and control sample in terms of total aflatoxin content. The total aflatoxin content of the control sample increased from 1.04 to 3.74 μg/kg (3.5 times) after four months of storage. In coated samples with 2% MC solution, the total aflatoxin content increased 1.5 times. On the other hand, there was no significant difference between coated samples with 0.1% and 0.5% of MC solution in terms of total aflatoxin content. Results showed that the total aflatoxin content of coated samples with 0.1% and 0.5% of MC solution was reduced significantly during the storage so that they could not be detected after four months of storage period. The reason can be traced back to moisture and temperature conditions that are two crucial factors for aflatoxin production; however, this research temperature was constant, but moisture content varied with different concentrations. The highest amount of moisture loss was related to the control sample, and the lowest one has belonged to pistachio kernel coated with 2% MC solution. It can be attributed to the effects of packaging type, storage in the incubator at a constant temperature, and coating agent, which acted as a good moisture insulator that leads to the lowest moisture loss during the storage period. Also, possible hydrophobic particles present in MC coating act as a barrier agent and reduced surface vapor, limited water migration, and retarded moisture loss during storage (Guimarães et al., 2020). On the other hand, the control sample showed an enhanced total of aflatoxins due to the lack of an appropriate coating compared with other treatments.

### 3.2 Yeast and mold count

Pistachio nuts coated with 0.1%, 0.5%, 1%, and 2% MC solution and control sample were exposed to microbial test at different time intervals (0, 2, and 4 months) of storage period. There was a significant difference between samples in terms of microbial tests (*p* <.05). The microbial load is equal to zero in coated samples with a 0.1%, 0.5%, 1%, and 2% MC solution (Table 2). Regarding the constant growth trend in coated samples, the ascending growth trend is only related to the control sample. It should be noted that mold growth in the control sample was limited due to the lack of oxygen and low packaging environment moisture content (Cardoso et al., 2017; Jafarzadeh et al., 2020). These results are consistent with Hashemi et al. (2021), which reported that the edible coating containing alginate and thyme oil reduced mold and yeast growth in fresh pistachio. Similarly, Razavi et al. (2021) claimed that the carboxymethyl cellulose coating enriched with *thymus vulgaris* extract decreased the yeast and mold count of hazelnut during 147 days of storage and the uncoated sample showed the highest amount of yeast and mold. Besides, Maghsoudlou et al. (2013) reported that the mold growth was almost zero in the pistachio nut samples that contained edible coating while it highly increased in the control sample during storage.

### 3.3 Total count

As shown in Table 2, the MC coating significantly affected the total count during the storage period (*p* < .05). The control sample had the highest microbial load, whereas coated samples showed the lowest microbial load (zero). As MC acts as active packaging material, which contains antimicrobial agents, it leads to reduce the microbial load or suppress the microbial growth (Berenji Ardestani et al., 2019). The mechanism of MC antimicrobial activity is due to the interaction of cationic polymolecules of MC with anionic components of the microorganism cell wall that resulted to the change in cell wall permeability (Bahrami et al., 2020). By this mechanism, some substances are maintained inside the cell and food materials are inhibited from entering into the cell. After entering into the cell and bonding with DNA, MC inhibits RNA synthesis, leading to microorganisms' cellular death (Agriopoulou et al., 2020). Another reason for antimicrobial activity is oxygen and moisture content reduction of packaging material due to the packaging type. When comparing pistachio nuts to Iran National Standard No. 218 (pistachio nut, properties and test method for yeast, mold, and total count), it becomes clear that these factors fall in permitted levels, indicating accuracy precision of test conditions.

### 3.4 Sensory attributes

**FIGURE 3:**
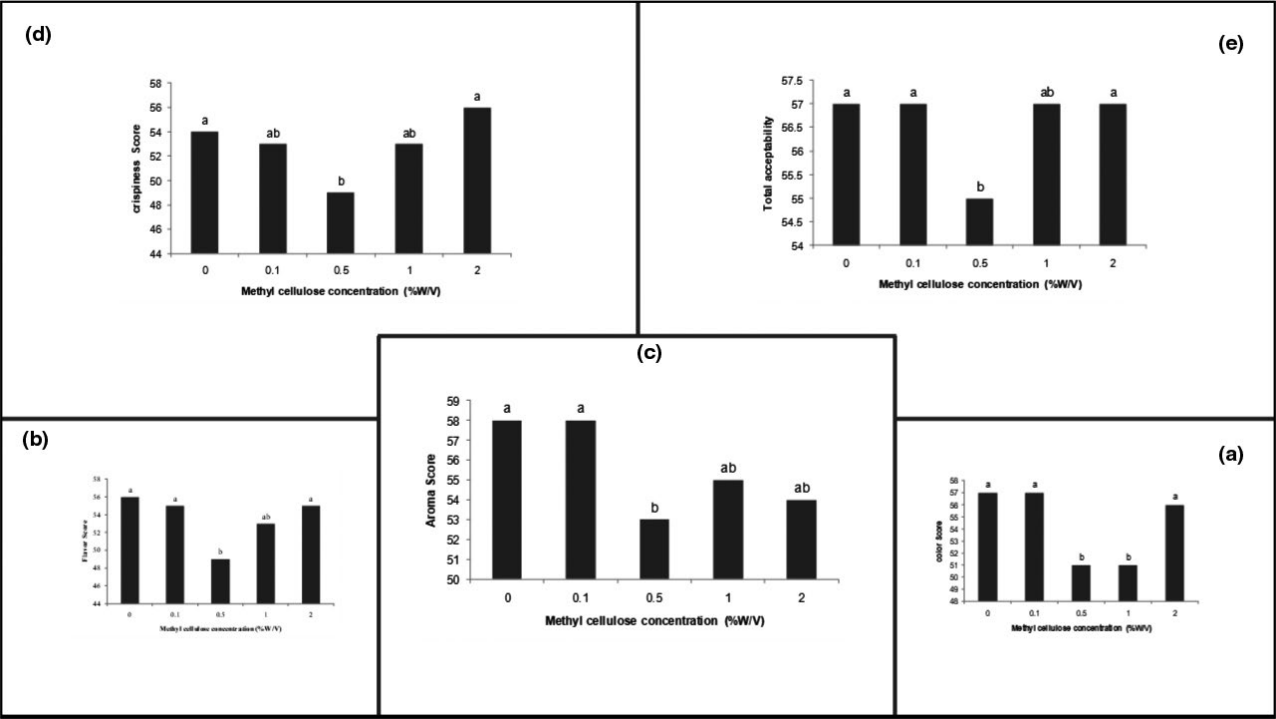
Assessor scores for (a) color, (b) flavor, (c) aroma, (d) crispiness, and (e) total acceptability of pistachio nut coated by different concentrations of methylcellulose

Sensory evaluation was performed based on a hedonic scale using 12 trained panelists. As can be seen from Figure 3a, coated pistachio nut samples with a 0.1% and 2% MC solution and a control sample had the highest color score, followed by coated samples with a level of 0.5% and 1% MC solution. In terms of color, no significant difference was observed between control and coated samples with a 0.1% and 2% MC solution.

In terms of flavor, a significant difference was observed between control and coated pistachio nut samples with 0.1%, 1%, and 2% MC (*p* < .05) (Figure 3b). However, there was no significant difference between coated samples with 0.5%, 1%, and 2% MC solution and control sample (*p* < .05).

There was no significant difference between control and coated nut pistachio samples with 0.1%, 1%, and 2% MC solution in terms of crispiness. However, there was a considerable difference between coated samples with 0.1% and 2% MC solution and control sample with coated sample with 0.5% MC. Regarding that the coated samples with 0.1%, 1%, and 2% MC are not different from the control sample, all of them had the best crispiness, followed by a coated sample with a 0.5% MC solution, which is not different from 0.1% and 1% MC solution‐coated samples (Figure 3c).

As can be seen from Figure 2d, no significant difference was observed between coated samples with 0.1%, 1%, and 2% MC solution and control sample in terms of aroma. However, there was a significant difference between coated samples with 0.1% and 0.5% MC solution and control sample (*p* < .05). Regarding that pistachio nuts coated with 0.1, 1, and 2% MC solution are not different from the control sample in terms of aroma, all of them are found to have the best aroma followed by the sample coated with 0.5% MC, which does not have a significant difference from coated samples with 1% and 2% MC.

In total acceptability, control and coated samples with 0.1%, 1%, and 2% MC get the highest scores, while the coated sample with 0.5% MC solution receives the lowest score by assessors (Figure 3e). Maghsoudlou et al. (2013) investigated the effect of chitosan coating on the color, flavor, texture, and general acceptability of pistachio nuts. The results showed an insignificant change in sensory properties of pistachio nuts with different concentrations of chitosan coating.

### 3.5 SEM characteristics of coated pistachio nuts

#### 3.5.1 Coating diameter

**FIGURE 4:**
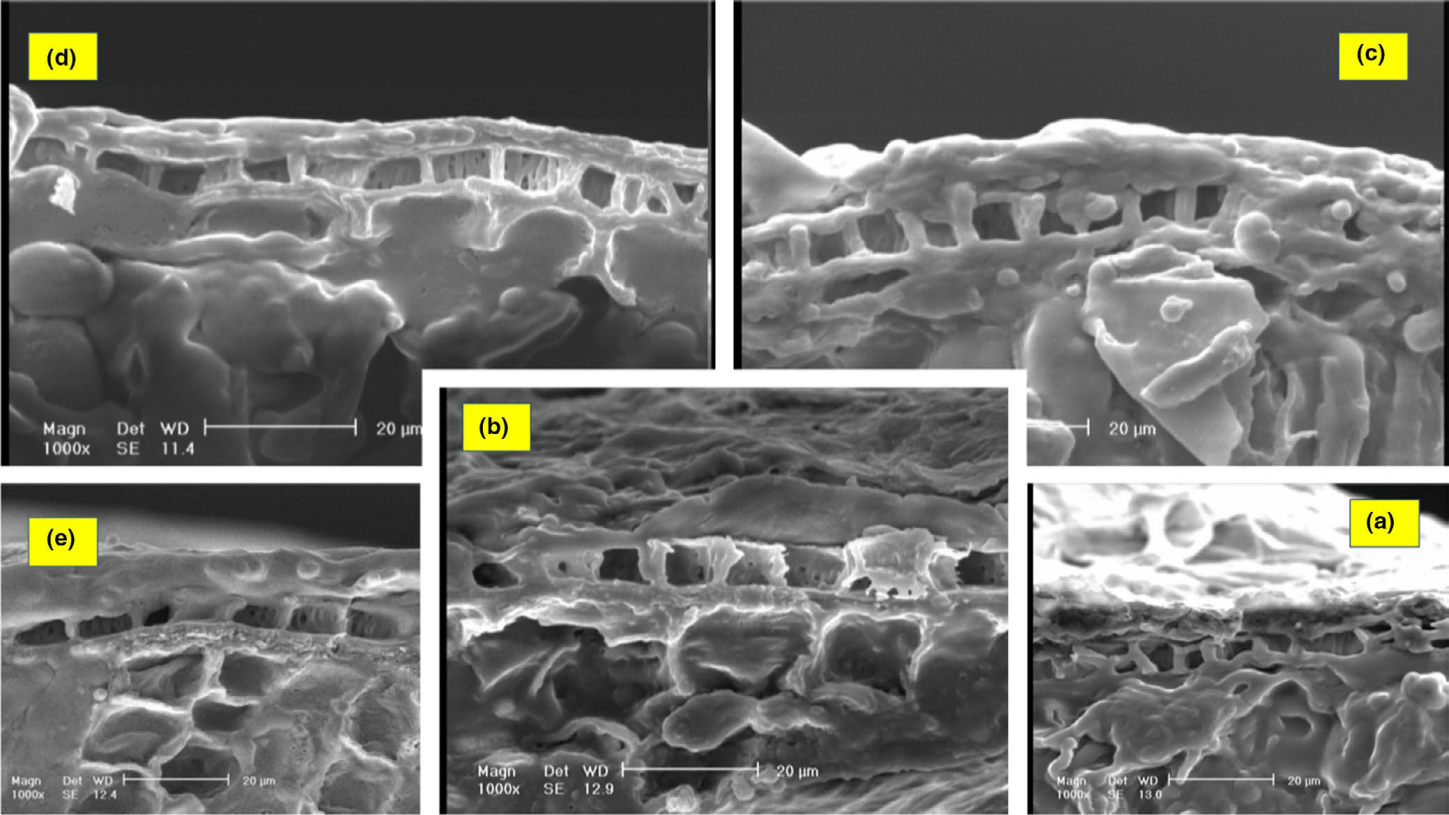
SEM images of pistachio cross section (a) Control sample, (b) sample coated with 0.1% methylcellulose, (c) sample coated with 0.5% methylcellulose, (d) sample coated with 1% methylcellulose, and (e) sample coated with 2% methylcellulose. The accuracy of the cross‐sectional images of the samples in the 1% and 2% concentrations coated with methylcellulose indicates that these samples' coatings have a higher uniformity than the other samples

Figure 4 indicates that all of the coatings have a good density. The coated sample with 1% MC has a lower diameter than the other samples, but it shows the highest density and viscosity. The reason for this low diameter is the high viscosity and density of layers at this concentration.

#### 3.5.2 Methylcellulose coating layer uniformity

**FIGURE 5:**
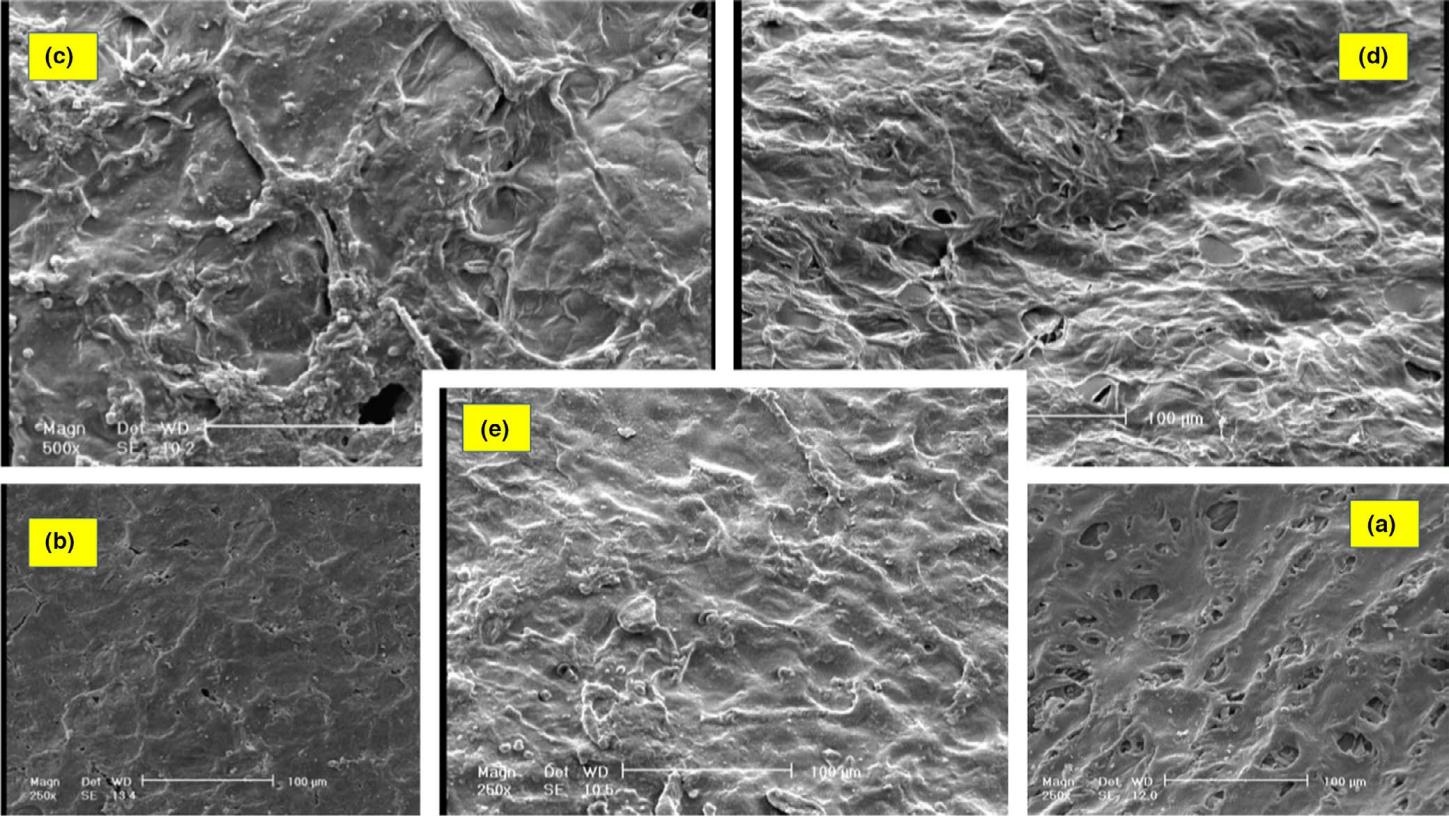
SEM images of pistachio surface (a) Control sample, (b) sample coated with 0.1% methylcellulose, (c) sample coated with 0.5% methylcellulose, (d) sample coated with 1% methylcellulose, and (e) sample coated with 2% methylcellulose

Coating uniformity can be evaluated by investigation of coating surface roughness. It should be mentioned that to obtain electron microscopic images, different parts of the surface and cross sections of samples were investigated. Analysis of the cross‐sectional surface indicates that the sample surfaces are thoroughly coated, and no fracture was found. As can be seen from Figure 5, the surface roughness of coated samples is different. Figure 5d,e is more uniformed than Figure 5b,c, and Figure 5e shows more uniformity than 5d. Examination of the images shows that all coatings have a good density. According to the comparison of the average values of samples, although the thickness of the sample cover is 0.5% less than other samples, by comparing the images, in terms of density and adhesion, the 2% sample has the highest density and adhesion compared with other samples.

## 4 CONCLUSION

In the present study, we successfully developed a MC edible coating and applied it to pistachio nut. The microbial, morphological, and sensory properties of coated pistachios were monitored during four months of storage. The results showed that the MC coating had a significant inhibitory effect on aflatoxin contamination. The best concentration of MC coating to reduce aflatoxin contamination and retain pistachio nut sensory attributes was found in 1% MC solution; however, the highest amount of aflatoxin contamination was related to the control sample (3.5%). All samples except coated samples with 0.5% MC solution had reasonable total acceptability. The experimental data suggest that the MC coating reduced aflatoxin, yeast, and mold growth in pistachio nuts compared with uncoated samples. Therefore, MC coating is recommended for keeping the quality of pistachio nuts. The appropriate coating of such valuable products (pistachio) is a good step for reducing aflatoxin amount, and enhancing shelf life and total acceptability from an export perspective.
